# Effects of carboplatin combined with paclitaxel-based intraperitoneal hyperthermic perfusion chemotherapy on serum levels of HE4 and DJ-1 in patients with advanced recurrent ovarian cancer

**DOI:** 10.12669/pjms.38.4.4736

**Published:** 2022

**Authors:** Xianhui Su, Xuewen Sun, Yanhui Kang, Yuna Dai

**Affiliations:** 1 Xianhui Su, Department of Pharmacology, Medical College of Hebei Engineering University, Handan, 056038, Hebei, China; 2 Xuewen Sun, Department of Pathogenic Biology, Medical College of Hebei Engineering University, Handan, 056038, Hebei, China; 3 Yanhui Kang, Department of Pharmacology, Medical College of Hebei Engineering University, Handan, 056038, Hebei, China; 4 Yuna Dai, Department of Breast Surgery, Affiliated Hospital of Hebei Engineering University, Handan 056029, Hebei,China

**Keywords:** Chemotherapy, Ovarian cancer cells, Tumor markers

## Abstract

**Objective:**

To analyze the effects of carboplatin combined with paclitaxel-based intraperitoneal hyperthermic perfusion chemotherapy (IPCH) on serum levels of human epididymis protein 4 (HE4) and mitogen-dependent oncogene-1 (DJ-1) in patients with advanced recurrent ovarian cancer (OC).

**Methods:**

From July 2019 to July 2020, patients with advanced recurrent OC (n=92) treated in Affiliated Hospital of Hebei Engineering University were selected as study subjects. According to the random number table method, patients were divided into control group and observation group. Patients in the control group were treated with carboplatin combined with paclitaxel-based intravenous chemotherapy, and patients in the observation group were treated with carboplatin combined with paclitaxel-based IPCH. The therapeutic effects, serum levels of HE4, DJ-1 and human kallikrein 10 (HK-10), peripheral blood immune indexes and adverse reactions of patients were compared between the two groups.

**Results:**

The response rate of the observation group was significantly higher than that of the control group (*p<*0.05); after treatment, the indexes of HE4, DJ-1 and HK-10 in the two groups were significantly decreased, while the indexes of CD3^+^CD4^+^, CD3^+^CD56^+^ and CD3^+^CD4^+^/CD3^+^CD8^+^ were significantly increased; moreover, significantly lower indexes of HE4, DJ-1 and HK-10 and significantly higher indexes of CD3^+^CD4^+^, CD3^+^CD56^+^ and CD3^+^CD4^+^/CD3^+^CD8^+^ were found in the observation group when compared with the control group (*p*<0.05). The severity of myelosuppression, nausea and vomiting in the observation group was significantly lower than that in the control group, and the total tumor metastasis rate in the observation group was significantly lower than that in the control group (*p<*0.05).

**Conclusions:**

Carboplatin combined with paclitaxel IPCH had obvious inhibitory effects on HE4, DJ-1 and other serum tumor markers in patients with advanced recurrent OC, with a more prominent clinical effect, and could further significantly reduce the risk of adverse reactions and metastasis.

## INTRODUCTION

Ovarian cancer (OC), a malignant tumor growing in ovarian tissues, was more common in gynecologic malignancies. According to the clinical treatment experience^1^,^2^, it has been found that the morbidity of OC was second only to cervical cancer and endometrial cancer. However, the mortality of OC was the highest among gynecologic malignancies, resulting in serious adverse effects on women’s physical health and safety. In recent years, surgery has been believed as the main clinical plan for the treatment of OC, supplemented by chemotherapy. Surgical treatment could remove the lesion tissues to the maximum extent and reduce the burden for subsequent chemotherapy.^3^,^4^ In addition, targeted therapy, immunotherapy, traditional Chinese medicine treatment and other methods also had certain improvement effects in the recovery process of OC patients, but with limited research data, thus continuous analysis and exploration were still necessary.^5^,^6^ Among the chemotherapy drugs for OC, carboplatin, a broad-spectrum anti-tumor drug, had almost no cross resistance, high drug activity and low risk of gastrointestinal adverse reactions and nephrotoxicity, with certain clinical application value, but targeted nursing should be performed against myelosuppression and other side effects. Paclitaxel was extracted from a natural plant--Taxus chinensis, with the main anti-cancer component being diterpenoid alkaloids, and paclitaxe played a key role in the treatment of OC patients.^7^,^8^ Generally, conventional chemotherapy was administered through the intravenous route. A relevant study has proposed that, for patients with advanced recurrent OC, intraperitoneal hyperthermic perfusion chemotherapy (IPCH) could further improve the treatment efficiency and the quality of life of patients.^9^ In this study, ninety-two patients with advanced recurrent OC were selected to be performed with intravenous chemotherapy and IPCH by grouping, the recovery of different patients was analyzed, and the report was submitted as follows:

## METHODS

Patients with advanced recurrent OC (n = 92) treated in Affiliated Hospital of Hebei Engineering University from July 2019 to July 2020 were selected as the study subjects.

### Ethical approval:

The study was approved by the Institutional Ethics Committee of Affiliated Hospital of Hebei Engineering University, and written informed consent was obtained from all participants.

### Inclusion criteria:

- Patients with recurrent epithelial OC (clinical stage, III-IV) who were confirmed according to the relevant diagnostic criteria^10^ and pathological and cytological examination results;
- Patients without other surgical operations performed in the past six months;
- Patients who agreed to voluntarily participate in this study;
- Patients with perfect clinical records.
- Exclusion criteria:
- Patients diagnosed with neuropsychiatric disease or cognitive impairment;
- Patients diagnosed with severe organic disease or physical disability;
- Patients in pregnancy or lactation;
- Patients combined with other malignant tumors.

According to the random number table method, the enrolled patients were divided into two groups: the control group (n = 46) and the observation group (n = 46). Patients in the control group were 30-60 years old, with an average age of 44.59 ± 1.33 years old; as for tumor typing, there were 27 cases of serous adenocarcinoma, 10 cases of endometrioid carcinoma and nine cases of mucinous adenocarcinoma; regarding clinical staging, there were 18 cases of stage IIIa, 16 cases of stage IIIb, 12 cases of stage IV. Patients in the observation group was 32-64 years old, with an average age of 45.06 ± 5.28 years old; as for tumor typing, there were 26 cases of serous adenocarcinoma, 11 cases of endometrioid carcinoma and 9 cases of mucinous adenocarcinoma; regarding clinical staging, there were 16 cases of stage IIIa, 17 cases of stage IIIb and 13 cases of stage IV. There was no significant difference in general information between the two groups (*p>*0.05).

### Methods:

Patients in both groups received conventional treatment, such as diuretic therapy, hydration treatment, prevention and treatment of vomiting, etc., and received anti-anaphylactic treatmentt, such as intramuscular injection of diphenhydramine (Shanghai zhaohui Pharmaceutical Co., Ltd., SFDA approval number: H31021087) one hour before chemotherapy. (1) Patients in the control group was treated with carboplatin combined with paclitaxel chemotherapy; drugs: Carboplatin Injection (Bristol-Myers Squibb S.R.L., SFDA approval number: H20171063, 150 mg/tube) and Paclitaxel Injection (Bristol-Myers Squibb S.R.L., HJ20171227, 30 mg/5ml); methods: intravenous drip of Paclitaxel Injection was carried out, with the specification being 175 mg/m^2^, d1; intravenous drip of Carboplatin Injection was performed, with the specification being 5.6 AUC, d1; and treatment cycle: once per 28 days;

Patients in the observation group were treated with carboplatin combined with paclitaxel IPCH, catheterization was carried out in the abdominal cavity of patients (the catheterization site was in the central venous vessel), and drugs and normal saline (1,500 ml, about 45°C) were infused through the catheter, the specifications of Paclitaxel Injection and Carboplatin Injection were 75 mg/m^2^ and 150 mg/m^2^, respectively; duration of hyperthermic perfusion: 1 hour per time; frequency of position conversion: 20 min/time, 3-4 times in total (conducive to uniform drug distribution); and treatment cycle: once per seven days and then suspended for seven days after three consecutive sessions. The study cycle was 3 months.

### Observation indexes

The treatment effects were compared between the two groups, and Immune-Modified Response Evaluation Criteria In Solid Tumors (imRECIST)^11^ was used as the evaluation tool. The specific contents were: complete remission (all the target lesions of patients were eliminated), partial remission (the maximum diameter reduction rate of the target lesions of patients was 20%-30% after treatment), disease progression (the maximum diameter growth rate of the target lesions of patients was 20%-30% after treatment), disease stabilization (the disease state of patients was between partial remission and disease progression), and the response rate = (complete remission + partial remission) cases/total cases × 100%.

The serum tumor markers, including human epididymal protein 4 (HE4), mitogen-dependent oncogene-1 (DJ-1), human kallikrein 10 (HK-10), were compared between the two groups before and after treatment; test methods: fasting venous blood (5 ml) samples were collected, after conventional centrifugal separation, HE4, DJ-1 and HK-10 were determined by double-antibody sandwich enzyme-linked immunosorbent assay (ELISA)

The peripheral blood immune indexes were compared between the two groups before and after treatment, including CD3^+^CD4^+^, CD3^+^CD56^+^and CD3^+^CD4^+^/CD3^+^CD8^+^; methods: fasting peripheral venous blood (5 ml) samples were collected from patients, and CD3^+^CD4^+^, CD3^+^CD56^+^, CD3^+^CD4^+^/CD3^+^CD8^+^ and other indicators were measured by Attune NxT flow cytometry.

The adverse reactions were compared between the two groups after treatment, with the main observation objects being myelosuppression and nausea and vomiting symptoms, and the severity of adverse reactions was divided into 0-IV grade according to the RTOG/EORTC criteria^12^; and tumor metastasis status was compared between the two groups after treatment.

### Statistical Analysis:

SPSS21.0 software was selected to analyze the relevant data of the study. The enumeration data was expressed as rate (%), X^2^ test was performed, and ranked data was analyzed by rank-sum test; while the measurement data was expressed as mean ± standard deviation (x̄± s), and *t* test was performed. P*<*0.05 was considered as statistically significant.

## RESULTS

The response rate of the observation group was significantly higher than that of the control group (*p<*0.05) (Table-I).Before treatment, there was no significant difference in HE4, DJ-1 and HK-10 between the two groups (*p>*0.05); after treatment, the indexes of HE4, DJ-1 and HK-10 in the two groups were significantly decreased, and the indexes of HE4, DJ-1 and HK-10 in the observation group were significantly lower than those in the control group (*p<*0.05) (Table-II).

**Table-I:** Comparison of treatment effects between patients in the observation group and the control group (n, %).

Group	Cases	Complete remission	Partial remission	Disease progression	Disease stabilization	Response rate
Control group	46	6	14	15	11	20 (43.48)
Observation group	46	8	22	9	8	30 (65.22)
X^2^						4.381
P						0.036

**Table-II:** Comparison of serum tumor markers between the two groups before and after treatment (x¯ ± s).

Group	Cases	HE4 (pmol/L)		DJ-1 (μg/L)		HK-10 (μg/L)	

		Before treatment	After treatment	Before treatment	After treatment	Before treatment	After treatment
Control group	46	235.08 ± 10.22	45.98 ± 3.15	14.59 ± 1.34	10.38 ± 1.01	13.57 ± 1.78	10.72 ± 1.07
Observation group	46	236.12 ± 11.01	38.12 ± 5.76	14.61 ± 1.31	8.95 ± 1.14	13.60 ± 1.81	8.61 ± 1.02
t		0.470	8.120	0.072	6.368	0.080	9.681
P		0.640	0.000	0.943	0.000	0.936	0.000

Before treatment, there was no significant difference in CD3^+^CD4^+^, CD3^+^CD56^+^ and CD3^+^CD4^+^/CD3^+^CD8^+^ between the two groups (*p>*0.05); after treatment, the indexes of CD3^+^CD4^+^, CD3^+^CD56^+^ and CD3^+^CD4^+^/CD3^+^CD8^+^ in the two groups were significantly increased, and the observation group had significantly higher indexes of CD3^+^CD4^+^, CD3^+^CD56^+^ and CD3^+^CD4^+^/CD3^+^CD8^+^ when compared with the control group (*p<*0.05) (Table-III).The severity of myelosuppression and nausea and vomiting in the observation group was significantly lower than that in the control group (*p<*0.05) (Table-IV).The total tumor metastasis rate of the observation group was significantly lower than that of the control group (*p<*0.05) (Table-V).

**Table-III:** Comparison of peripheral blood immunity indexes between the two groups before and after treatment (x¯ ± s).

Group	Cases	CD3^+^CD4^+^		CD3^+^CD56^+^		CD3^+^CD4^+^/CD3^+^CD8^+^	

		Before treatment	After treatment	Before treatment	After treatment	Before treatment	After treatment
Control group	46	29.23 ± 3.19	35.12 ± 3.16	6.05 ± 1.03	6.98 ± 1.06	1.05 ± 0.04	1.53 ± 0.05
Observation group	46	29.25 ± 3.22	45.01 ± 3.09	6.04 ± 1.09	8.01 ± 1.07	1.06 ± 0.03	2.19 ± 0.12
t		0.030	15.177	0.045	4.638	1.356	34.433
P		0.976	0.000	0.964	0.000	0.178	0.000

**Table-IV:** Comparison of adverse reactions between the two groups after treatment (n,%).

Adverse reactions	Staging	Control group (n= 46)	Observation group (n= 46)	Z	p
Myelosuppression	0	5 (10.87)	15 (32.61)	22.529	0.000
	I	6 (13.04)	20 (43.48)		
	II	19 (41.30)	8 (17.39)		
	III	12 (26.09)	3 (6.52)		
	IV	4 (8.70)	0 (0.00)		
Nausea and vomiting	0	1 (2.17)	7 (15.22)	28.959	0.000
	I	7 (15.22)	27 (58.70)		
	II	20 (43.48)	9 (19.57)		
	III	13 (28.26)	2 (4.35)		
	IV	6 (13.04)	1 (2.17)		

**Table-V:** Comparison of tumor metastasis status between two groups after treatment (n, %).

Group	Cases	Hepatic metastases	Peritoneal metastasis	Pulmonary metastasis	Metastasis to other tissues	Total metastasis rate
Control group	46	4	4	5	3	16 (34.78)
Observation group	46	1	2	2	2	7 (15.22)
X^2^						4.696
P						0.030

## DISCUSSION

The fact of no obvious early clinical symptoms and the lack of clinical indicators with high sensitivity and specificity were suggested as the main reasons for the low rate of early diagnosis of OC in relevant patients, thus delaying the best treatment timing and increasing the physiological pain, economic burden and life risk of patients.^13^ At present, the relevant academic world have not fully elucidated the cause of OC, and the existing disease data showed that there was a high correlation between genetic factors and epithelial OC. In addition, it has been demonstrated that continuous ovulation and environment may also be high-risk pathological factors of OC.^14^ Generally, patients with malignant tumors of ovary would receive comprehensive treatment plans, that is, surgery combined with chemotherapy were applied to eliminate the lesion cells and prolong the survival of patients.^15^ For patients with advanced recurrent epithelial OC, cytoreductive surgery was more likely to be chosen, followed by chemotherapy with the combination of carboplatin, paclitaxel and other drugs. Different chemotherapy methods showed certain differences in the physiological recovery of patients.^16^ In order to explore a more efficient chemotherapy protocol, the physiological indexes of patients with advanced recurrent epithelial OC were compared and analyzed through performing intravenous chemotherapy and IPCH by grouping.

The results of our study showed that the response rate of the observation group was significantly higher than that of the control group, while the total tumor metastasis rate of the observation group was significantly lower than that of the control group, indicating that carboplatin combined with paclitaxel IPCH was more helpful to control the disease progression of advanced recurrent epithelial OC and reduce the risk of metastasis; and a previous study of Kuittinen et al.^17^ also supported our results. Wherein the pharmacological action of carboplatin was to destroy the normal components of DNA molecules and force the double coiled spiral structure to disintegrate, thus playing a role in inhibiting the proliferation of tumor cells.^18^

Paclitaxel could improve the secretion level of cytoplasmic microtubule dimer in the body and block the mitotic process of tumor cells. Moreover, it has been reported that carboplatin combined with paclitaxel could enhance the inhibitory effect on tumor cells.^19^ IPCH, a combination of hyperthermia and local chemotherapy, could not only improve the sensitivity of chemotherapy drugs to tumor cells and reduce the risk of metastasis and recurrence of tumor cells, but also further increase the drug content in the liver tissue and reduce the risk of liver metastasis because the administration site was in the abdominal cavity with shorter distance to inferior vena cava and hepatic portal vein. Compared with intravenous chemotherapy, IPCH could ensure the utilization rate of chemotherapy drugs in the lesion site to a greater extent and improve the drug efficacy.^20^,^21^

Comparative analysis on the serum tumor markers of patients were carried out, and the results showed that, after treatment, the indexes of HE4, DJ-1 and HK-10 in the two groups decreased significantly, suggesting that the two chemotherapy methods may be helpful in the treatment of patients with advanced recurrent epithelial OC. However, the indexes of HE4, DJ-1 and HK-10 in the observation group were significantly lower than those in the control group, revealing that carboplatin combined with paclitaxel IPCH could improve the elimination rate of tumor cells. Furthermore, decreased secretion levels of serum tumor markers were observed in patients. HE4 could reflect ovarian cancer more sensitively, and had certain reference value in the diagnosis and prognosis evaluation of related diseases. In addition, abnormally increased concentration of HE4 in the body indicated that there was increased risk of tumor-associated diseases.

Riggs et al.^21^ also pointed out that HE4 was positively correlated with age, and the concentration of HE4 was continuously increased with age. DJ-1, a member of the peptidase C56 protein family, could prevent neurons from oxidative stress and death. At the same time, as a specific protein expressed in epithelial OC cells, DJ-1 could more sensitively reflect the proliferation status of tumor-related cells, thus providing an evaluation basis with reference value for evaluate the therapeutic effect in OC patients.^22^.^23^ As reported by a previous study, there was a positive correlation between HK-10 and the progression of OC cells, namely, the more serious the progression of OC cells, the higher the concentration of HK-10 in the body.^24^

In addition, our study also found that the indexes of CD3+CD4+, CD3+CD56+ and CD3+CD4+/CD3+CD8+ in the two groups were significantly increased, indicating that the immune cell activity of patients was constantly improved, which was related to the decrease of malignant tumor cells, thus confirming the effectiveness of the two treatment plans. However, the indexes of CD3+CD4+, CD3+CD56+ and CD3+CD4+/CD3+CD8+ in the observation group were significantly higher than those in the control group, further proving that the efficacy of carboplatin combined with paclitaxel IPCH was more significant.These findings were partly consistent with the conclusion of a related study.^25^ The chemotherapy drugs had certain cytotoxicity, after analyzing the adverse reactions of the two groups, the results showed that the severity of myelosuppression and nausea and vomiting in the observation group was significantly lower than that in the control group, demonstrating that IPCH could reduce the cytotoxicity of chemotherapy drugs and reduce the incidence of side effects, so that the safety was more guaranteed.

### Limitations of the study

More data may be obtained by stratified design according to body mass index; the study had a small sample size, short follow-up period and no more detailed subgroup comparison. Our findings were still needed to be further confirmed by more in-depth studies in the future.

## CONCLUSION

For patients with advanced recurrent OC, carboplatin combined with paclitaxel IPCH could significantly reduce the secretion levels of serum tumor markers such as HE4 and DJ-1, enhance the therapeutic effect and reduce the risk of metastasis and the incidence of side effects, with a better clinical application prospect.

### Authors’ Contributions:

**XS and XS** designed this study and prepared this manuscript,and are responsible and accountable for the accuracy or integrity of the work.

**YD** collected and analyzed clinical data.

**YK** significantly revised this manuscript.
